# Hypoxia Induces Saturated Fatty Acids Accumulation and Reduces Unsaturated Fatty Acids Independently of Reverse Tricarboxylic Acid Cycle in L6 Myotubes

**DOI:** 10.3389/fendo.2022.663625

**Published:** 2022-03-11

**Authors:** Lukas Vacek, Ales Dvorak, Kamila Bechynska, Vit Kosek, Moustafa Elkalaf, Minh Duc Trinh, Ivana Fiserova, Katerina Pospisilova, Lucie Slovakova, Libor Vitek, Jana Hajslova, Jan Polak

**Affiliations:** ^1^ Department of Pathophysiology, Third Faculty of Medicine, Charles University, Prague, Czechia; ^2^ Institute of Medical Biochemistry and Laboratory Diagnostics, First Faculty of Medicine, Charles University, Prague, Czechia; ^3^ Institute of Food and Nutrition Analysis, Faculty of Food and Biochemical Technology, University of Chemistry and Technology in Prague, Prague, Czechia; ^4^ Department of Physiology, Faculty of Medicine in Hradec Králové, Charles University, Hradec Králové, Czechia; ^5^ 4^th^ Department of Internal Medicine, Faculty General Hospital and 1^st^Faculty of Medicine, Charles University, Prague, Czechia

**Keywords:** hypoxia, reverse TCA, L6 myotubes, glutamin, lipids, obstructive sleep apnea

## Abstract

Obstructive sleep apnea syndrome, characterized by repetitive episodes of tissue hypoxia, is associated with several metabolic impairments. Role of fatty acids and lipids attracts attention in its pathogenesis for their metabolic effects. Parallelly, hypoxia-induced activation of reverse tricarboxylic acid cycle (rTCA) with reductive glutamine metabolism provides precursor molecules for *de novo* lipogenesis. Gas-permeable cultureware was used to culture L6-myotubes in chronic hypoxia (12%, 4% and 1% O_2_) with ^13^C labelled glutamine and inhibitors of glutamine uptake or rTCA-mediated lipogenesis. We investigated changes in lipidomic profile, ^13^C appearance in rTCA-related metabolites, gene and protein expression of rTCA-related proteins and glutamine transporters, glucose uptake and lactate production. Lipid content increased by 308% at 1% O_2,_ predominantly composed of saturated fatty acids, while triacylglyceroles containing unsaturated fatty acids and membrane lipids (phosphatidylcholines, phosphatidylethanolamines, phosphatidylinositol) decreased by 20-70%. rTCA labelling of malate, citrate and 2-hydroxyglutarate increased by 4.7-fold, 2.2-fold and 1.9-fold in 1% O_2_, respectively. ATP-dependent citrate lyase inhibition in 1% O_2_ decreased lipid amount by 23% and increased intensity of triacylglyceroles containing unsaturated fatty acids by 56-80%. Lactate production increased with hypoxia. Glucose uptake dropped by 75% with progression of hypoxia from 4% to 1% O_2_. Protein expression remained unchanged. Altogether, hypoxia modified cell metabolism leading to lipid composition alteration and rTCA activation.

## Introduction

Obstructive sleep apnea syndrome (OSA) is a chronic disease affecting 5 – 15% of the general population (1), manifesting with partial airway narrowing or complete occlusion during sleep with subsequent sleep fragmentation and decreased blood oxygen levels. OSA was recently identified as one of the risk factors for development of cardiovascular diseases (2) and Type 2 diabetes mellitus (3), however, mechanisms linking OSA with impaired glucose metabolism remains unknown. Insulin sensitivity and glucose uptake in muscle attracts particular attention, as skeletal muscle is responsible for 75-80% of glucose disposal (4) after meals and together with adipose tissue, pancreatic β-cells and liver participate in glucose homeostasis.

Despite intermittent nature of rapid oxygen desaturations observed in arterial blood of OSA patients, tissue oxygen levels follow a different pattern defined by local tissue oxygen consumption and tissue blood flow (5). In fact, direct muscle tissue oxygen measurements in mice exposed to intermittent hypoxia (mimicking severe OSA) showed a steady hypoxic O_2_ levels (6) motivating thus our choice of sustained (rather than intermittent) hypoxic exposure in the present study, to better model pathophysiological processes associated with OSA in muscle.

Reverse TCA (rTCA), also known as reverse Krebs cycle, represents a phylogenetically preserved metabolic pathway observed in prokaryotic organisms as well as eukaryotic cancer cells, liver, heart and brown adipose tissue (7–9). Reverse TCA represents a uniform response of mitochondria to reduced respiratory chain activity, as typically observed under hypoxia or chemical inhibition of respiration (10). Reduced O_2_ availability inhibits mitochondrial electron flux through respiratory chain and increases thus NADH/NAD^+^ ratio in mitochondria. To prevent arrest of the Krebs cycle, mitochondrial transhydrogenase transfer electrons from NADH to NADP^+^ forming NADPH (11) which can be subsequently used inside mitochondria or transported to cytoplasm where it is used as a reductive agent in *de novo* fatty acid synthesis. Reactions in rTCA, where 2-oxoglutarate is reduced and carboxylated to citrate, are catalyzed by isocitrate dehydrogenase 2 and aconitase. Glutamine provides anaplerotic substrate as it enters rTCA after deamination to glutamate and subsequently to 2-oxoglutarate (12). Citrate is subsequently converted to oxaloacetate and acetyl-CoA by ATP-dependent citrate lyase, generating thus acetyl-CoA as a substrate for *de novo* lipogenesis (13).

Excessive accumulation of intracellular fatty acid and lipids (like ceramides and diacylglycerols) was shown to impair insulin signaling and glucose uptake in skeletal muscle *via* multiple mechanisms, including PKB inhibition, induction of serine 307 phosphorylation for IRS-1 or activation of PKC or NFκB (14). Increased cellular adiposity (e.g. hepatic steatosis) represents an established response to hypoxia (15), however sources and composition of intracellular lipids remain to be elucidated. Previous studies reported that hypoxia-exposed myotubes showed decreased free fatty acids (FFA) uptake and oxidation (16), suggesting the role of intracellular *de novo* lipogenesis, presumably *via* rTCA, providing both: acetyl-CoA as a building block and NADPH as a reductive agent for fatty acid synthesis. Furthermore, plasma free fatty acids are often elevated in OSA patients (17), which can be partially attributed to increased adipose tissue lipolysis (13, 18, 19), however, the role of *de novo* lipogenesis in various cell types should also be considered.

The aim of this study was to determine the impact of pericellular oxygen levels on rTCA activation, intracellular lipid and fatty acid quantity and composition combined with a functional assessment of glucose uptake and protein/gene expression of key proteins in L6-myotubes (cell line derived from rat skeletal muscle). To achieve this goal, we employed gas-permeable cultureware allowing long-time exposure to sustained predetermined levels of pericellular oxygen levels (20, 21) and exposed cells to three different O_2_ levels representing: control exposure (12% O_2_, modelling aortic O_2_ levels), mild hypoxia (4% O_2_, modelling physioxic skeletal muscle tissue O_2_ levels (22) and severe hypoxia (1% O_2_, modelling skeletal muscle tissue O_2_ levels expected in patients with OSA (6).

Specific inhibitors of enzymes catalyzing entrance of glutamine to rTCA or *de novo* lipogenesis, as well as ^13^C labelled glutamine were employed and combined with state-of-the-art lipidomic and selected gene/protein expression analysis.

Our results show that exposure to severe hypoxia increases intracellular lipids containing saturated FA, partially through activation of rTCA, as well as total lipid amount, while protein expression of glutamine transporters, glutaminase and ATP-dependent citrate lyase remained unchanged. Compared to 4% O_2_ glucose uptake was reduced in hypoxic (1% O_2_) as well as hyperoxic conditions (12% O_2_), however, this effect is not mediated by activation of rTCA.

## Materials and Methods

### L6-Myotubes Cell Culture, Differentiation and Hypoxic Exposure

Myoblasts (L6-C11, rat skeletal muscle cells; European Collection of Cell Cultures, Cat. No. 92102119) were cultured in Dulbecco’s Modified Eagle’s Medium (DMEM, Cat. No. D6429, Sigma Aldrich, USA) supplemented with 10% fetal bovine serum (FBS, Cat. No. F6178, Sigma Aldrich, USA), 1% Penicillin-Streptomycin (Cat. No. P4333, Sigma Aldrich, USA) and 1% HEPES (Cat. No. H0887, Sigma Aldrich, USA). At passage No. 10, cells were plated into 24-well fluorocarbon-bottom dishes (Cat. No. 94.6000.014, Sarstedt AG & Co, Germany) or 50mm fluorocarbon-bottom wells (Cat. No. 94.6077.410, Sarstedt AG & Co, Germany) at the initial density of 2800 cells/cm^2^ and cultured with media exchange every 2 days in the CO_2_ incubator at 37°C until reaching confluence (7 days). Subsequently, FBS concentration was reduced to 2% (promotion of spontaneous differentiation) and cells were placed into the modular hypoxic incubator (Billups-Rothenberg Inc., USA) where mild and severe hypoxic exposures were achieved by inflating the incubators with calibration-quality gas mixtures of 1% O_2_ + 5% CO_2_, 4% O_2_ + 5% CO_2_ and 12% O_2_ + 5% CO_2_ (Linde Gas a.s., Czech Republic). Hypoxic exposures lasted 7 days, while media were exchanged every 2 days and differentiation was confirmed by microscopic observation of characteristic myotubes structures. Subsequently, cells were used for the experiments described below. Representative microscopic photographs of differentiated and undifferentiated cells are provided in the **Supplementary Material Figures 3A, B** together with gene expression of GLUT1/GLUT4 transporters in **Figure 4**.

### Treatments With Chemical Compounds

Drugs dissolved in DMSO (stock solution) were added to culture media for last 24 hours, while for control treatments, identical amounts of DMSO in culture media was used. Concentration of drugs as used in this study was based on previously published data (13, 23, 24). The following chemicals were used: 1 mM aminooxyacetic acid hemihydrochloride (AOA, 4-aminobutyrate aminotransferase inhibitor, Cat. No. C13408, Sigma Aldrich, USA) and 2 μM bis-2-(5-phenylacetamido-1,3,4-thiadiazol-2-yl)ethyl sulfide (BPTES, glutaminase inhibitor, Cat. No. SML0601, Sigma Aldrich, USA); 500 nM N-[5-[4-[6-[[2-[3-(trifluoromethoxy)phenyl]acetyl]amino]-3-pyridazinyl]butyl]-1,3,4-thiadiazol-2-yl]-2-pyridineacetamide (CB-839, glutaminase 1 inhibitor, Cat. No. 22038, Cayman Chemicals, USA) and 40 μM (3R,5S)-*rel*-5-[6-(2,4-dichlorophenyl)hexyl]tetrahydro-3-hydroxy-2-oxo-3-furanacetic acid (SB-204990, citrate lyase inhibitor, Cat. No. 15245, Cayman Chemicals, USA). The concentration of 40 μM for SB204990 was chosen based on previous reports showing sufficient inhibition of ACLY in a cell line and to closely mimic plasma concentrations achieved in animal experiments after oral administration (25).

### Intracellular Total Lipid Content Measurement

Total lipids were extracted using Lipid Extraction Kit (Cat. No. 211044, Abcam, UK). Briefly, cells were washed with PBS and lysate collected into Lipid Extraction Buffer. Subsequently, lipids were extracted and quantified using fluorescent dye specifically labelling lipids, following manufacturer’s instructions (Lipid Assay Kit (Cat. No. 242307, Abcam, UK). Data were normalized to protein concentration in lysates quantified by the bicinchoninic acid method (Cat. No. 23227, ThermoFisher Scientific, USA).

### Lipidomic Analysis

Details of the lipidomic analysis can be found in the dataset accompanying this paper.

### Fatty Acid Methyl Esters Analysis (FAMES)

After the complete cultivation, cells were lyophilised, and lipids were extracted to mixture of methyl *tert*-butyl ether and methanol. The esterification reaction was performed with methanol with 0,5M sodium hydroxide under catalysing of boron trifluoride at 80°C. Subsequently, esters were extracted to heptane. The identification was performed by gas chromatography with flame ionization detector (GC-FID), where retention times of standards and sample peaks were compared. The analysis was performed on Agilent 6890N Network Gas Chromatograph with SP-2560 column. The temperature programme was 140°C for 5 minutes, 4°C/minute to 240°C and 240°C for 15 minutes. Injection volume - 1 ul, injection temperature - 240°C, detector temperature - 260°C. As mobile phase was used helium on 1,1 ml/min flow and the time of acquisition was 45 minutes.

### Reverse Tricarboxylic Acid Cycle (rTCA) Activation Analysis

Cells were exposed to medium containing 2.5mM ^13^C glutamine labelled on C1 position (Cat. No. CLM-3612-PK, Cambridge Isotope Laboratories, Inc., USA) or unlabeled glutamine (control experiments) for 24 hours. Subsequently, medium was removed, plates were washed by cold PBS and dishes were immediately placed on dry ice blocks. The frozen cells were scraped and lysed in the mixture of chloroform, distilled water and methanol (2:1:1). This suspension was vigorously shaken, vortexed and finally centrifuged (3000 RPM/10 min). The upper polar phase containing our analytes was evaporated under the stream of nitrogen (65°C) or lyophilized over-night, and then derivatized with chlorotrimethylsilane/*N,O*-Bis(trimethylsilyl)acetamide/pyridine 1/2/4 v/v/v. These samples were directly injected into GC/MS. GC/MS was employed to analyze labelled metabolites using an Agilent 6890 instrument coupled to an Agilent 5973 mass spectrometer and Agilent ChemStation software (Agilent Technologies, Palo Alto, CA). The ratios between ^13^C fragments and ^12^C fragments were calculated with corrections for a background, determined from the non-labelled samples, in which for malate, 2-hydroxyglutarate, citrate and glutamate the derivatized fragmented ions m/z 335, 349, 273 and 345. Thus for incorporation into malate, ^13^C-labeled malate ions 336 were traced vs. ions 335, 2-hydroxyglutarate-originating ions 350 vs. ions 349, citrate-originating ions 274 vs. ions 273, as well as glutamate-originating ions 349 were compared with ^12^C glutamate ions 348. For assessment of influence of hypoxia on rTCA-related metabolites production, the concentrations of 12C malate, 2-hydroxyglutarate, citrate, glutamate and 2-oxoglutarate was measured and normalized on protein concentration and internal standard.

### Reverse Tricarboxylic Acid Cyle (rTCA) Contribution to Fatty Acid Synthesis

The preparation of samples for analysis of incorporation of C5 labelled glutamine to fatty acids was different. Cells were exposed to medium containing 2.5mM ^13^C glutamine labelled on C5 position (Cat. No. CLM-1822-H-PK, Cambridge Isotope Laboratories, Inc., USA) or unlabeled glutamine (control experiments) for whole hypoxic exposure (7 days). The cell pellets washed with PBS were extracted with water/methanol/choroform (1:1:2, v/v/v) and centrifuged at 1,000 x g for 10 min. The lower nonpolar phase was transferred into a glass vial and dried under nitrogen gas. Lipid hydrolysis was performed with ethanolic KOH by redissolving samples in 80% ethanol with 0,5 mM potassium hydroxide and incubated at 60°C for 20 min. The lipids were extracted with ethanol/hexane (2:1, v/v) after neutralization with acetic acid. The upper nonpolar phase was transferred into a vial glass, dried under nitrogen gas, and samples were derivatized with diazomethane at room temperature for 20 min. After drying, the derivatized samples were redissolved in 200 µL of hexane and directly injected into a gas chromatograph – mass spectrometer. The column used for this analysis was RESTEK Rxi-5ms (15 mx0,25 mm IDx0,25µm) and the mobile phase (He) rate was set at 1 mL.min^-1^. The injector temperature was 310°C and the injected volume was 5 µL. The oven program started with 1 min 100° C hold, then increased at 10°C/min to 250°C and to final temperature 310°C at 20°C/min. The MS operated in SIM mode and set m/z were: 270, 271, 272, 273 and 274. As a representation of fatty acid pool, we chose palmitate – the most common result of *de novo* fatty acids. Results were expressed as the % incorporation of ^13^C to total carbon amount of palmitate.

### The 2-Deoxyglucose Uptake Determination

Prior to glucose uptake determination using Glucose Uptake-Glo™ Assay kit (Cat. No. J1343, Promega, USA), cells were incubated in serum-free and glucose-free media for 24 hours. Subsequently, the cells were washed with PBS and incubated with 0.1 mM 2-deoxyglucose for 30 minutes and 2-deoxyglucose-6-phosphate quantified after 120-min incubation using a luminometer with 1 second integration time (Infinite^®^ 200 PRO, Tecan, Switzerland), according to manufacturer’s instructions.

### Determination of Lactate Production

During lactate production determination, cells were incubated in serum-free and lactate-free media and samples of culture media were collected after 24 hours. The lactate concentration in culture media was determined using colorimetric method with the L-Lactate Assay Kit (Cat. No. ab65330 Abcam, USA) according to manufacturer’s instructions.

### Gene and Protein Expression Analysis

qPCR: Total RNA was isolated and treated with DNAse using High Pure RNA Isolation Kit (Cat. No. 11828665001, Roche Diagnostics, Switzerland). Subsequently, cDNA was transcribed with High-capacity cDNA Reverse Transcription Kit (Cat. No. 4368814, Roche Diagnostics, Switzerland) and gene expression of ATP-dependent citrate lyase (ACLY), glutaminase 1 (GLS), glutamine transporters SLC38A2 and SLC1A5, β-glucuronidase (GUSB) and TATA box binding protein (TBP) was assessed using quantitative PCR with TaqMan probes (Product ID: Rn00566411_m1, Rn00561285_m1, Rn00710421_m1, Rn00598400_m1, Rn00566655_m1, Rn01455646_m1, Applied Biosystems, Carlsbad, CA) and Real Time PCR cycler ABI 750 (ThermoFisher Scientific, USA). Data were presented as relative gene expression (compared to TBP and GUSB reference genes) using the 2^-ΔΔCt^ method.

Western blotting: Cells cultured in 55mm dishes were lysed in 1mL T-PER lysis buffer (Cat. No. 78510, ThermoFisher Scientific, USA) and centrifuged (10.000 rpm, 10 min, 4°C) to separate supernatant. Proteins in supernatants were separated after mixing with Laemmli buffer (1:1, Cat. No. 161-0737, Bio-Rad Laboratories, USA) using SDS/PAGE electrophoresis in 8% or 10% gels and then blotted onto a 0.2 μm PVDF membrane for 1 h at 100 V in precooled Transfer buffer (10x diluted, Cat. No. 2317, Bio-Rad Laboratories, USA). The membranes were blocked with 5% BSA (A2058, Sigma-Aldrich, USA) in TBS-T buffer (100 mM Tris-HCl, 150 mM NaCl, pH = 7.6, 0.1% Tween-20) for 60 min. After washing with TBS-T, membranes were incubated with the primary antibody overnight on the shaker placed in the refrigerator, subsequently washed in TBS-T, incubated for 1 h with the secondary antibody (1:10.000, goat anti-rabbit IgG conjugated with a horseradish peroxidase, sc-2004, Santa Cruz Biotechnology (Dallas, Texas, USA) and bands detected using enhanced chemiluminescence method with Radiance PLUS Chemiluminescent Substrate (Azure Biosystems, USA) and digitalized with ChemiDoc Imaging System (Bio-Rad, USA). Densitometric analysis was performed in using Image Lab software (Bio-Rad, USA). Band intensities of all proteins were normalized to β-tubulin signal. The following primary antibodies were used: ATP-dependent citrate lyase (1:2000, Abcam, ab40793), Glutaminase (1:500, ThermoFisher Scientific, Prod. No. 701965), SLC1A5 (1:1000, ThermoFisher Scientific, PA5-88700), SLC38A2 (1:1000, Abcam, ab90677) and β-tubulin (1:5000, Abcam, ab6046) was performed by western blot analysis.

### Statistical Analysis

The effect of hypoxic exposures on mean values in studied variables was analyzed using one-way ANOVA, while interaction between hypoxia and chemical treatments was assessed using 2-way ANOVA in GraphPad Prism 8 software (GraphPad Software, USA). Data are presented as the mean ± SEM. A value of p < 0.05 was considered to reflect a statistically significant difference in all tests.

All lipidomic datasets (initially and after each variable reduction step) were preprocessed in the same fashion before employing statistical analysis (total ion current normalized, Log transformed, and Pareto scaled). Principal Component Analysis (PCA) score plots in Metaboanalyst was used for data overview. The first filtration step was ANOVA for lipid significance (α = 0.01) on lipids between three groups with various oxygen level performed in Metaboanalyst. Only lipids with false discovery rate (FDR) p-value <0.01 were retained in the data matrix. This reduced dataset was imported to SIMCA (Soft Independent Modelling of Class Analogies; Sartorius, Germany) where Partial Least Squares Discriminant Analysis (PLS-DA) was performed with the goal to separate the groups. The rule to assess the lipid significance in this analysis was its PLS-DA VIP (variable importance in projection) score > 1. Hierarchical clustering analysis using Ward clustering algorithm and Euclidean distances options (HCA) was performed in Metaboanalyst to view clusters of significant lipids. This further reduced final dataset was subjected to calculate fold changes between groups and Spearman correlation coefficients with a specific intensity pattern. T-test was used to evaluate the effect of inhibitor presence with significance level α=0.01.

## Results

### The Effect of Hypoxia and ATP-Dependent-Citrate-Lyase Inhibitor on Intracellular Lipid Content

**Figure 1:**
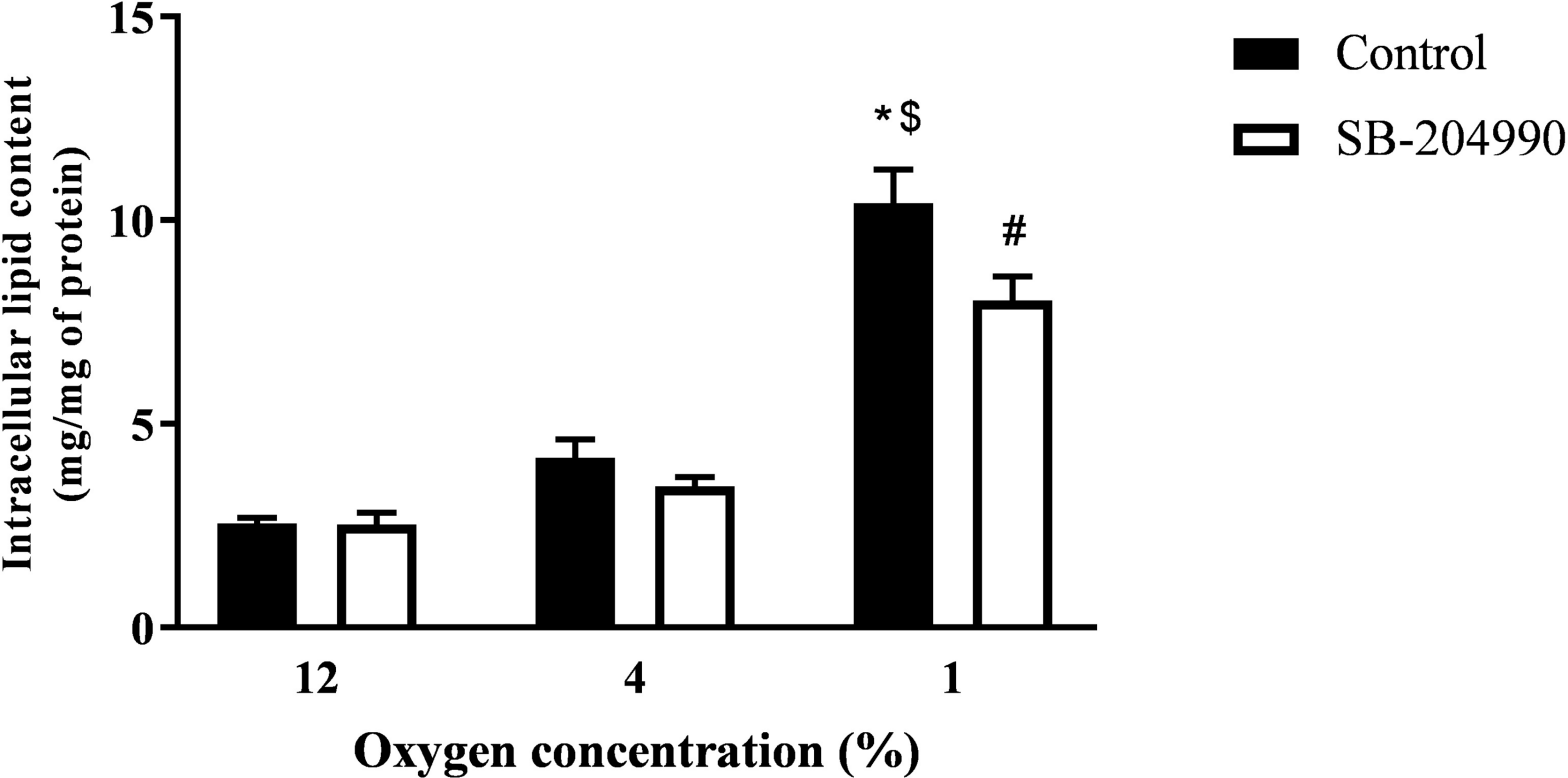
The Effect of Hypoxia and ACLY Inhibitor on Intracellular Lipid Content. The effect of hypoxia and ACLY inhibitor (SB-204990) on lipid accumulation in cells. Differentiated L6 myotubes were exposed to hypoxia for 7 days, subsequently treated with 40 μM SB-204990 for 24 hours and lipid content data compared to respective vehicle-treated (DMSO) cells. N = 6; ^*^p < 0.01 for comparison with control exposures (12% O_2_, 2-way ANOVA); ^$^p < 0.01 for comparison with 4% O_2_ exposures (2-way ANOVA); ^#^p < 0.05 for comparsion with vehicle treated cells (2-way ANOVA).

Exposure to 4% O_2_ and inhibition of ATP-dependent citrate lyase had no effect on intracellular lipids. However, increasing the severity of hypoxic exposure to 1% O_2_ not only augmented intracellular lipid amount to 308% of control exposure (2.55 ± 0.15 *vs.* 10.42 ± 0.82 mg lipids/mg proteins, p < 0.01), but also revealed a contribution of citrate to *de novo* lipid synthesis as documented by a 23% drop in lipids after treatment with ATP-dependent citrate lyase inhibitor SB-204990 (10.42 ± 0.82 *vs.* 8.03 ± 0.60 mg lipids/mg proteins, p < 0.05), as displayed in **Figure 1**.

### The Effect of Hypoxia on Intracellular Lipidome - Response to Hypoxic Exposures

**Figure 2:**
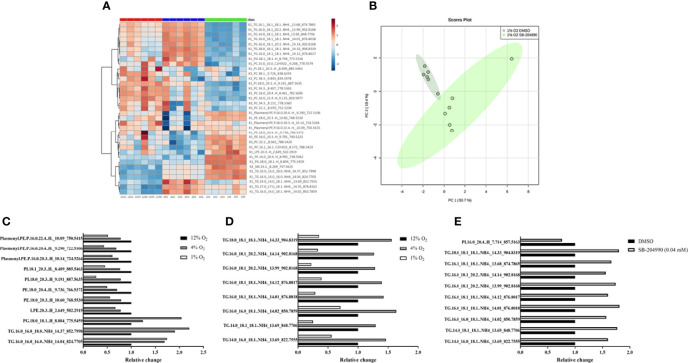
The Effect of Hypoxia on Lipidomic Profile. The effect of hypoxia on intracellular lipidomic profile based on ANOVA FDR p-value < 0.01 and PLS-DA VIP score > 1. Data are presented as HCA map with colorful visualization of log_2_FC (Fold change) – **(A)**. The effect of hypoxia and ATP-dependent citrate lyase inhibition (40 μM SB-204990) on clustering of cell samples in 1% O_2_. Data are presented as PCA Score plot **(B)**. Lipids with increased intensity with progression of hypoxia (**C**, p < 0.01). Lipids with “up and down” pattern (increased at 4_%_ O2, decreased at 1% O_2_) (**D**, p < 0.01). Lipids affected by ACLY inhibitor at 1% O_2_ (**E**, p < 0.01).

Lipidomic analysis identified 164 lipid compounds in cellular lysates, of which 35 molecules showed significant differences among hypoxic exposures based on ANOVA FDR p-value < 0.01 and PLS-DA VIP score > 1. A dose response increase by 30% and 104% with exposures to progressive hypoxia was observed only for phosphatidylglycerol (containing 18:0 and 18:1 FA), while triacylglyceroles containing palmitate and stearate showed hypoxia-induced increase irrespective of its severity. In contrast, lipid compounds contained in cellular membranes e.g. lysophosphatidylethanolamine and phosphatidylethanolamines (C16-20:3), phosphatidylcholines (C16-C22 with saturated and unsaturated FA), phosphatidylinositol (containing saturated and unsaturated C18-20 FA) and plasmalogens (C16-C24) were decreased in hypoxic exposures by 20-70% predominantly in a dose-response manner. Data are summarized in HCA map (**Figure 2A**), graph with discussed lipid molecules (**Figure 2C**) and **Supplementary Material No.1**.

A distinct “up and down” pattern (increase under 4% O_2_ with subsequent decrease in 1% O_2_ compared to control exposures) was observed for unsaturated FA (C18:1 and C20:2) contained in 8 various triglyceride molecules. Interestingly, based on lipidomic analysis, none of significantly different lipid molecules showed “down and up” pattern (decrease under 4% O_2_ with subsequent increase in 1% O_2_ compared to control exposures). Data are summarized in HCA map (**Figure 2A**), graph with discussed lipid molecules (**Figure 2D**) and **Supplementary Material Table 1**, **2**.

### The Effect of Hypoxia on Intracellular Lipidome - Response to ATP-Dependent Citrate Lyase Inhibition

Chemical inhibition of ATP-dependent citrate lyase with 40 µM SB-204990 under severe hypoxia, based on t-test FDR p-value < 0.01, increased appearance of unsaturated FA containing one or two unsaturated bonds (C16:1, C18:1 and C20:2) found in 9 triacylglyceroles by 56-81%, and decreased content of phosphatidylinositol (C16_C20:4) by 32%, suggesting role of citrate (and subsequent release of acetyl-CoA) for intracellular synthesis of these lipid molecules under severely hypoxic conditions. Importantly, ATP-dependent citrate lyase inhibitor showed no impact on lipid profile under control and mild hypoxic conditions. Data are summarized in PCA Score plot (**Figure 2B**), graph with discussed lipid molecules (**Figure 2E**) and **Supplementary Material Table 1**, **2**.

### Fatty Acid Methyl Esters Analysis - FAMES

There was detected 51 signals during the gas chromatography analysis. 22 of them were identified thanks to fatty acid methylesters standards. Seven fatty acids were somehow affected by hypoxia. Lauric acid (C12:0) increased by 106% at 4% O_2_ and by 506% at 1% O_2_, myristic acid (C14:0) by 60% at 1% O_2_, stearic acid (C18:0) by 21% at 1% O_2_, linolelaidic acid (C18:2n6t) by 46% at 1% O_2_, behenic acid (C22:0) by 42% at 1% O_2_ and lignoceric acid (C24:0) 180% at 1% O_2_. Only palmitoleic acid (C16:1c) was decreased at severe hypoxia by 30%.

There was also observed the effect of ACLY inhibitor. At 1% O_2_ C12:0, C14:0 and γ-linolenic acid (C18:3n6) were decreased after SB-204990 treatment by 60%, 14% and 30%, respectively. At 4% O_2_ elaidic acid (C18:1n9t) and C18:2n6t were increased by 38% and 31%, respectively. At 12% O_2_ C16:1c, oleic acid (C18:1n9c) and C18:2n6t were decreased by 29%, 8% and 7%, respectively, and C22:0 was increased by 22%. Data are summarized in the **Table 1**.

**Table 1:** The Effect of Hypoxia and ACLY inhibitor on Intracellular Fatty Acid Composition.

FA	12% O_2_ (vehicle)	12% O_2_ (inhibitor)	4% O_2_ (vehicle)	4% O_2_ (inhibitor)	1% O_2_ (vehicle)	1% O_2_ (inhibitor)
C12:0	0.16 ± 0.02	0.18 ± 0.09	0.33 ± 0.03^*^	0.28 ± 0.03	0.97 ± 0.08^*#^	0.58 ± 0.06^$^
C14:0	2.25 ± 0.07	2.08 ± 0.13	2.31 ± 0.14	2.43 ± 0.09	3.60 ± 0.12^*#^	3.02 ± 0.14^$^
C15:0	0.35 ± 0.03	0.19 ± 0.10	0.36 ± 0.04	0.27 ± 0.02	0.24 ± 0.02	0.11 ± 0.06
C16:0	12.32 ± 0.17	12.77 ± 0.49	12.48 ± 0.61	12.21 ± 0.16	14.64 ± 0.45	13.04 ± 0.66
C16:1c	3.72 ± 0.37	2.64 ± 0.09^$^	3.69 ± 0.18	3.13 ± 0.28	2.59 ± 0.32^*#^	2.14 ± 0.20
C17:0	0.39 ± 0.05	0.21 ± 0.11	0.38 ± 0.03	0.41 ± 0.02	0.53 ± 0.04	0.38 ± 0.07
C18:0	12.24 ± 0.73	13.71 ± 0.59	11.99 ± 0.61	11.29 ± 0.13	14.78 ± 0.24^*^#^^	14.67 ± 0.44
C18:1n9c	17.92 ± 0.31	16.45 ± 0.25^$^	17.31 ± 0.81	18.76 ± 0.45	19.73 ± 0.56	18.05 ± 0.87
C18:1n9t	0.28 ± 0.03	0.21 ± 0.10	0.26 ± 0.03	0.36 ± 0.01^$^	0.54 ± 0.10	0.35 ± 0.04
C18:2n6c	0.15 ± 0.09	0.12 ± 0.06	0.07 ± 0.08	0.11 ± 0.05	0.41 ± 0.08	0.34 ± 0.03
C18:2n6t	1.06 ± 0.00	0.99 ± 0.02^$^	0.89 ± 0.01	1.17 ± 0.08^$^	1.55 ± 0.23^*^#^^	1.67 ± 0.04
C18:3n6	0.71 ± 0.09	0.53 ± 0.12	0.63 ± 0.05	0.86 ± 0.06	0.76 ± 0.08	0.53 ± 0.01^$^
C20:2	0.04 ± 0.04	0.00 ± 0.00	0.00 ± 0.00	0.04 ± 0.03	0.11 ± 0.05	0.12 ± 0.06
C20:3n6	0.22 ± 0.06	0.16 ± 0.08	0.16 ± 0.08	0.23 ± 0.04	0.12 ± 0.06	0.13 ± 0.06
C20:4n6	6.59 ± 0.84	7.81 ± 0.04	6.23 ± 0.01	5.77 ± 0.17	5.79 ± 1.21	7.76 ± 0.12
C20:5n3	0.31 ± 0.04	0.37 ± 0.07	0.16 ± 0.08	0.24 ± 0.02	0.33 ± 0.03	0.23 ± 0.12
C21:0	0.00 ± 0.00	0.00 ± 0.00	0.00 ± 0.00	0.00 ± 0.00	0.07 ± 0.07	0.05 ± 0.05
C22:0	0.57 ± 0.00	0.69 ± 0.01^$^	0.50 ± 0.06	0.51 ± 0.00	0.81 ± 0.13^*^#^^	0.95 ± 0.02
C22:5n3	1.53 ± 0.12	1.49 ± 0.16	1.42 ± 0.05	1.43 ± 0.09	1.86 ± 0.16	1.84 ± 0.14
C22:6n3	1.59 ± 0.13	1.60 ± 0.26	1.49 ± 0.09	1.38 ± 0.03	1.82 ± 0.23	1.88 ± 0.03
C24:0	0.05 ± 0.05	0.00 ± 0.00	0.00 ± 0.00	0.00 ± 0.00	0.14 ± 0.08^*^#^^	0.25 ± 0.05
C24:1n9	0.17 ± 0.02	0.06 ± 0.06	0.11 ± 0.06	0.16 ± 0.09	0.15 ± 0.08	0.20 ± 0.03

The average percentage of particular identified fatty acids (FA) in the cells. 40 μM SB-204990 was used as an inhibitor of ATP-dependent citrate lyase, DMSO in appropriate amount was used as a vehicle. Data are displayed as average percentage ± SEM. ^*^p < 0.05 for comparison with vehicle exposures (12% O_2_ DMSO, ANOVA FDR). ^#^p < 0.05 for comparsion with 4% O_2_ DMSO exposures (ANOVA FDR). ^$^p < 0.05 for comparison with DMSO exposures (T-Test). N = 3 for all test.

### The Effect of Hypoxia on rTCA Activation

**Figure 3:**
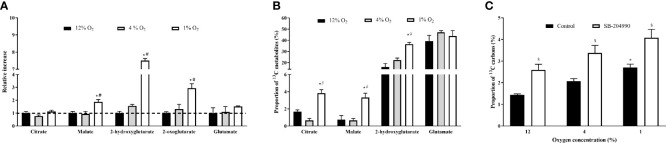
The Effect of Hypoxia on Incorportaion of 13C to rTCA Metabolites and Fatty Acids. The effect of hypoxia on quantity of metabolites generated in rTCA **(A)**. Incoporation of ^13^C derived form 1-^13^C-labelled glutamine to metabolites generated in rTCA, subtracted from the effect of non-labelled glutamine **(B)** and percentage of ^13^C carbon derived from 5-^13^C-labelled glutamine in intracellular palmitate **(C)**. ^*^p < 0.05 for comparison with 12% O_2_ (ANOVA, **A** - N = 3, **B, C** – N = 6); ^#^p < 0.05 for comparsion with 4% O_2_ exposures (ANOVA, **A** - N = 3, **B, C** – N = 6); ^$^p < 0.05 for comparsion with DMSO - Control (ANOVA, N = 6).

Metabolite labelling through rTCA under all conditions was investigated using the stable-isotope labelled glutamine, containing ^13^C on the C1 position entering the rTCA as 2-oxoglutarate and being sequentially reductively carboxylated to citrate. Exposure to 1% O_2_ but not 4% O_2_, elevated absolute quantity of malate by 88% (1.00 ± 0.12 *vs.* 1.88 ± 0.17, p < 0.01), 2-hydroxyglutarate by 650% (1.00 ± 0.14 *vs.* 7.50 ± 0.13, p < 0.01) 2-oxoglutarate by 194% (1.00 ± 0.11 *vs.* 2.94 ± 0.34, p < 0.01), while citrate levels remained unchanged, **Figure 3A**. Furthermore, severe but not mild hypoxia increased incorporation of ^13^C from glutamine to citrate by 2.2-fold (1.7 ± 0.2 *vs.* 3.8 ± 0.4, p < 0.01), to malate by 4.7-fold (0.7 ± 0.4 *vs.* 3.3 ± 0.4, p < 0.01) and to 2-hydroxyglutarate by 1.9-fold (19.2 ± 0.9 *vs.* 36.5 ± 1.6%, p < 0.01), **Figure 3B**.

### The Effect of Hypoxia and ACLY Inhibition on 13C Incorporation Into Palmitate

The tracing of glutamine to fatty acid molecules *via* rTCA was conducted by measurement of palmitate containing ^13^C carbon originating form C5 labelled glutamine. In comparison to 12% O_2_, exposure to 1% O_2_ increased palmitate ^13^C incorporation from 1.4% to 2.7% (p < 0.05), while the presence of ACLY inhibitor increased the incorporation of ^13^C to palmitate from 1.4% to 2.6% for 12% O_2_, from 2.1% to 3.4% for 4% O_2_, and from 2.7% to 4.1% for 1% O_2_ (p < 0.05). Data are summarized at **Figure 3C**.

In parallel, exposure to 1% and 4% O_2_ increased incorporation of ^13^C to citrate by compared 12% O_2_ from 1.0% to 1.9% for 4% O_2_ and from 1.0% to 2.1% for 1% O_2_ (p < 0.05). Administration of SB-204990 prevented hypoxia-induced ^13^C incorporation to citrate. At 4% O_2_ the incorporation decreased by from 1.9% to 1.0% and at 1% O_2_ by from 2.1% to 1.0% (p < 0.05).

### The Effect of Hypoxia on Lactate Production and Glucose Uptake

**Figure 4:**
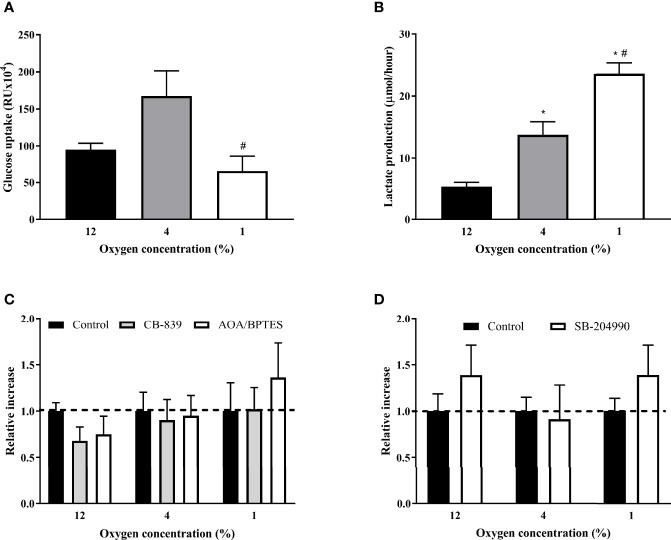
The Effect of Hypoxia and rTCA Inhibitors on Glucose Uptake and Lactate Production. The effect of hypoxia **(A)**, glutamine mitochondrial transport inhibitors **(C)** and ATP-dependent citrate lyase inhibition **(D)** on glucose uptake or lactate production **(B)**. **(A, B)** Glucose uptake and 24-h lactate production was measured after exposure to hypoxia for 7 days. Differentiated L6 myotubes were exposed to hypoxia for 7 days and subsequently treated with 500 nM CB-839, 1 mM AOA, 2 μM BPTES **(C)** or 40 μM SB-204990 **(D)** for 24 hours. ^*^p < 0.05 for comparison with control exposures (ANOVA for **A**, **B** or 2-way ANOVA for **C, D**, N = 6); ^#^p < 0.05 for comparsion with 4% O_2_ exposures (ANOVA, N = 6).

The hypoxic exposure progressively increased lactate production by 159% and 344% in 4% O_2_ and 1% O_2_ (13.73 ± 2.13 and 23.53 ± 1.76 μmol/hour, respectively) compared to the control exposure (5.30 ± 0.71 μmol/hour, both p < 0.01), **Figure 4A**. Cells exposed to 1% O_2_ showed reduced glucose uptake by 75% (662.60 ± 201.60 *vs.* 167.54 ± 33.79 RUx10^4^, p < 0.05) compared to cells cultured in mild hypoxia (4% O2), resulting in glucose uptake similar to control conditions. Glucose uptake at mild hypoxia was only non-significantly enhanced by 76% (95.13 ± 8.67 *vs.* 167.54 ± 33.79 RUx10^4^, p = 0.1), **Figures 4A, B**. This effect of severe hypoxia was not mediated by modified glutamine entrance to rTCA as inhibiting glutamine to 2-oxoglutarate conversion by specific inhibitors (AOA/BPTES or CB-839) did not modify glucose uptake under any experimental condition. Additionally, CB-839 treatment did not modify lactate production or intracellular lipid content (**Supplementary Material Figures 1A, B**) Furthermore, glucose uptake was not modified after SB-204990 (1.00 ± 0.14 *vs.* 1.39 ± 0.32, p = 0.9), pointing towards additional mechanisms other than rTCA derived citrate/acetyl-CoA in the regulation of hypoxia-induced impairments in glucose uptake, **Figures 4C, D**.

### The Effect of Hypoxia on Gene and Protein Expression

**Figure 5:**
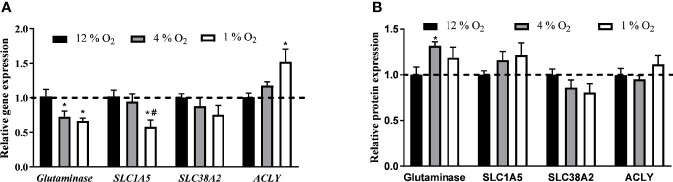
The Effect of Hypoxia on Expression of Genes and Proteins Involved in Glutamine Transport and Lipid Synthesis. The effect of hypoxia on gene **(A)** and protein **(B)** expression. Differentiated L6 myotubes were exposed to hypoxia for 7 days and gene and protein expression analysis was quantified. ^*^p < 0.05 for comparison with control exposures (12% O_2_, ANOVA, N = 6), ^#^p < 0.05 for comparison with 4% O_2_ exposures (ANOVA, N = 6).

Glutaminase was the only protein (among those we tested) whose gene expression was affected by 4% O_2_, specifically by 28% decrease (1.02 ± 0.10 *vs.* 0.73 ± 0.08 2^-ΔΔCt^, p < 0.05). Distinct transcriptional effects were observed in cells exposed to 1% O_2_ as demonstrated by 36 % decrease in *glutaminase* (1.02 ± 0.10 *vs.* 0.67 ± 0.04 2^-ΔΔCt^, p < 0.05), 44% decrease in *SLC1A5* (glutamine plasma membrane transporter; 1.02 ± 0.09 *vs.* 0.58 ± 0.10 2^-ΔΔCt^, p < 0.05) and, in contrast, 52% increase in *ACLY* (1.01 ± 0.06 *vs.* 1.52 ± 0.18 2^-ΔΔCt^, p < 0.05) gene expression, **Figure 5A**. Under 4% O_2_, among tested proteins only the glutaminase protein expression was stimulated by 32% (100.0 ± 8.5 *vs.* 131.8 ± 4.2%, p < 0.05). Despite observed changes in gene expression, modifications in pericellular O_2_ levels did not affect protein levels of investigated proteins, **Figure 5B**. Representative examples of western blot membranes are provided in the **Supplementary Material Figures 2A–D**.

## Discussion

In the present study, we investigated whether impaired glucose homeostasis observed in patients suffering from OSA (3) and modelled by experimental hypoxic exposures could be mediated by the direct effect of hypoxia in muscle. Specifically, using L6 myotubes *in vitro*, we explored whether mild and severe hypoxia modulated glucose uptake and whether such changes could be explained by quantity or quality (type, structure, class) of intracellular lipids. Furthermore, we employed stable ^13^C-labelled glutamine isotope to selectively trace reverse tricarboxylic acid cycle (rTCA) metabolites and to investigate the role of reductive glutamine metabolism. We observed that severe hypoxia (1% O_2_) reduced glucose uptake and enhanced accumulation of triacylglyceroles predominantly composed of saturated fatty acids, while diminished unsaturated FA. In parallel, we demonstrated increased activation of rTCA under severe hypoxia, however, based on experiments using selective inhibitors, only minor contribution of rTCA to intracellular lipid pool was observed under 1% O_2_. Observed effects are not mediated by modulation of glutamine entrance to rTCA or by expression of glutamine transporters.

Acute exposure to severe hypoxia has been shown to increase glucose uptake and impair insulin sensitivity (24, 25) through HIF-1 mediated transcriptional effects including regulation of lipid metabolism, e.g. increase in free fatty acid uptake (26) and *de novo* fatty acid synthesis (27) or decrease in fatty acid β-oxidation (28). In contrast, prolonged hypoxic exposures (multiple days), as performed in this study, were associated with normalization of HIF-1 levels (29) and modified cellular metabolism as a consequence of reduced oxygen availability. We have previously observed HIF-independent lipid accumulation in 3T3-L1 differentiated adipocytes exposed to hypoxia (13) and findings of unchanged protein expression of HIF-1 targets (glutaminase, SLC1A5, SLC38A2 and ACLY) (28–30) in the present study corroborated the notion of HIF-independent effects under chronic hypoxia. The activity of mitochondrial respiratory chain is strongly limited under severe hypoxia (31), however, alternative pathways for regeneration of reductive equivalents, like NADH, are employed to secure mitochondrial integrity and cell survival (32). Among these pathways, rTCA represents a well-established cell response to hypoxia utilizing reductive carboxylation of glutamine to produce citrate and subsequently acetyl-CoA – a key precursor metabolite for *de novo* lipid synthesis requiring reductive equivalents (33).

The ability of ^13^C-glutamine labelled on C1 position to specifically trace rTCA metabolites (as the ^13^C labelled carbon is lost as CO_2_ from carbon backbone during the forward TCA cycle) enabled us to quantify enrichment of citrate, malate and 2-hydroxyglutarate with labelled carbon to assess activation of rTCA under hypoxia. We observed that the overall rTCA metabolite labelling was doubled in L6-cells under severe hypoxia compared to control conditions, providing citrate as a building block for *de novo* lipogenesis (after conversion of citrate to oxaloacetate and acetyl-CoA). However, analyzing incorporation of ^13^C derived from glutamine labelled on C5 position (carrying this ^13^C all the way to acetyl-CoA and *de novo* synthesized lipids) to palmitate proved that hypoxia only minimally increased contribution of reductive glutamine metabolism to fatty acid synthesis (max 1.3% at 1% O_2_). Furthermore, rTCA contribution to *de novo* lipogenesis was not decreased after inhibition of ACLY. In fact, fractional incorporation of ^13^C (derived from 5-^13^C-glutamine) increased by few percentage points after ACLY treatment, which we explain by decreased quantity of unlabeled palmitate (and thus “paradoxical” increase in labelled palmitate carbons) in plasma membranes, phospholipids (phosphatidylcholines, plasmalogens, phosphatidylethanolamines) and by SB-204990 itself, as demonstrated by our lipidomic data (**Supplementary Material No.1**) as well as by other groups (34). Such observations extend previous findings of rTCA activation in cancer and other cell types (9, 12) and suggest the employment of this pathway also in differentiated cells, even though quantitative contribution of this pathways to total palmitate remains rather limited, as suggested by other authors showing very limited overall flux through rTCA pathways despite fatty acid labelling (35).

In parallel to activation of rTCA, exposure to severe hypoxia increased intracellular triacylglyceroles, which was only partially reduced by inhibition of ATP-dependent citrate lyase, a key factor for citrate conversion into acetyl-CoA. It needs to be emphasized, that *de novo* lipogenesis represents only minor contribution to total intracellular lipid pool under standard conditions with the predominant role of intracellular FFA transport through plasma membrane (36, 37). The majority of hypoxia-induced accumulation of lipids is thus probably mediated by increased FFA accompanied by 23% of rTCA contribution and possibly by other mechanisms e.g. mitochondrial dysfunction with reduced beta-oxidation (16, 26). Besides total triacylglycerole content, hypoxia also modified spectrum of fatty acids in triglycerides with predominant appearance of saturated FA such as palmitate and stearate under hypoxic condition, which is in line with established cellular responses to hypoxia known to pathologists as lipid dystrophy (hepatic steatosis, tiger heart) (15, 38). In contrast, unsaturated C16-18 FA were consistently downregulated by hypoxia which can be explained by reduced activity of SCD-1 (stearoyl-CoA desaturase 1) requiring oxygen as s acceptor of electrons in the desaturation reaction (39). When ACLY was inhibited under hypoxic conditions, intensity of unsaturated C16-18 FFA increased, corroborating thus the observation of hypoxia-induced synthesis of saturated FFA. To analyze the influence of hypoxia and ACLY inhibition on fatty acids, irrespectively of their intracellular origin and/or localization, we performed the FAMES (fatty acids methyl ester) analysis. In line with lipidomic observations, FAMES analysis showed upregulation of saturated fatty acids at severe hypoxia. In contrast, ACLY inhibition showed a predominantly suppressing effect on fatty acids intensity, while only unsaturated fatty acids at 4% O_2_ and C22 at 12% O_2_ were upregulated. However, it should be kept in mind, that lipidomic analysis and FAMES analysis represent two quite different approaches, first considering the biological nature of FA localized in various lipid compounds, while FAMES work with extracted FA from the whole cell.

The functional consequences of rTCA activation and increased lipid content with more saturated FFAs remain to be addressed. It was postulated previously, that acute hypoxic exposure rapidly increases the expression of several GLUT transporters (40) and glucose uptake (41). In line with these observations, glucose uptake was increased when L6-cells were chronically exposed to 4% O_2,_ however, no such effect was observed when cells were incubated under 1% O_2_. Based on our data, we speculate that chronic severe hypoxia (1% O_2_) is characterized by distinct metabolic features, including activation of rTCA, increased intracellular lipid content, and increased relative abundance of saturated FFA. However, based on presented results, it is not possible to conclude, whether these changes causally lead to reduced glucose uptake, or whether other mechanisms are involved. The hypoxic-induced stimulation of glucose uptake is mostly dependent on GLUT-1 glucose transporter, which expression is generally associated with chronic hypoxia (42). However, in the studies, that have already measured the expression of GLUT-1 as response to chronic hypoxia (in the model of impairing oxidative phosphorylation), the hypoxic exposure generally did not last more than 24 hours (43, 44). In our experiments, we exposed cells to severe hypoxia for seven days and there are no data to describe, whether GLUT-1 is overexpressed even after that long exposure. Such opposing effects of mild versus moderate hypoxic exposure were reported in the literature and might be associated with various degrees of initial HIF-1 activation or different impact on mitochondrial bioenergetics (45). Additionally, we demonstrated decreased glucose uptake while lactate production increased at 1% O_2_ exposures. We hypothesize, that oxaloacetate (formed after citrate lysis by ATP-dependent citrate lyase) together with malate (its metabolite) might be converted to pyruvate and subsequently to lactate as pyruvate was proved essential for maintaining cell metabolism under hypoxia – particularly due to its role in NAD^+^ homeostasis (46). Additionally, glucose for lactate production can be derived from intracellular glycogen stores (independently of glucose transport) present in L6 myotubes (47). The amount of glucose designated to pentose cycle is reduced in hypoxia in favor of glycolysis and lactate production (48). And finally, it is possible that reduced glucose uptake at 1% O_2_ provides sufficient amount of pyruvate for lactate production.

Limitations of the present study need to be acknowledged. First, this study investigated direct effects of pericellular O_2_ levels on functional aspects of L6-myotubes to model conditions of OSA, however, it should be noted that responses induced by hypoxia in the whole organism, including muscle tissue are more complex, involving endocrine changes (e.g. elevated concentrations of cortisol and catecholamines), production of reactive oxygen species and stimulation of inflammatory pathways (49). Second, employment of the membrane-bottom cultureware enabled exposure of cells to stable and defined pericellular oxygen levels during the whole experiment, however, we have previously reported modified protein expression profile induced by membrane material (50). Third, quantitative information about the contribution of rTCA pathway to *de novo* lipid synthesis would be possible using the 13C glutamine labelled on C5 position (51), however, this compound is currently unavailable from the manufacturer with unknown availability in the future and thus this task remains opened for future studies. Fourth, we measured glucose only relatively (in units of chemiluminescence), so we cannot quantify its production, which complicates the interpretation in relation to lactate production. Finally, selection of adequate O_2_ levels for *in-vitro* exposures represent a key factor in experimental design (5). In this study 12% O2 was considered as control as it the highest amount of oxygen achievable in human tissues (pulmonary veins contain 13% O_2_), 4% O_2_ represents mild hypoxia in this study is equivalent to physiological oxygen concentration in muscle tissue as measured in humans and rodents (4-5% O_2_) (6, 22) and 1% O_2_ represents a level of tissue oxygen achievable during apneic episodes in patients with severe OSA, based on direct measurements in rodent models (6).

In conclusion, exposure to severe hypoxia increased intracellular lipids containing saturated FA, partially through activation of rTCA, while protein expression of glutamine transporters, glutaminase and ATP-dependent citrate lyase remained unchanged. Compared to 4% O_2_ (representing physiological tissue O_2_ levels), glucose uptake was reduced in hypoxic (1% O_2_) as well as hyperoxic conditions (12% O_2_), however, this effect is not mediated by activation of rTCA. Direct tissue hypoxia thus partially contributes to impaired glucose homeostasis as observed in OSA probably through increased intracellular lipid stores and subsequent decrease of glucose uptake, with minor role of rTCA pathway.

## Data Availability Statement

The datasets presented in this study can be found in online repositories. The names of the repository/repositories and accession number(s) can be found below: https://www.ebi.ac.uk/metabolights/reviewer617a13a7-b437-4b9b-b978-3893b0d0f8ef.

## Author Contributions

LuV performed glucose uptake experiments, lactate and lipid assays, qPCR and Western Blot analysis and manuscript preparation. AD performed the experiments exploring the labelled glutamine incorporation. KB and VK performed lipidomic analysis. ME performed experiments with glucose uptake determination. MDT performed qPCR experiments and lactate analysis. IF performed Western blot analysis and lipid assay. KP, LS, and LiV conducted the experiments exploring the labelled glutamine incorporation. JH supervised and interpreted results of lipidomic analysis. The roles of JP were design, supervision and coordination of the study, data analysis and manuscript preparation. All authors have participated in the manuscript preparation and approved its final form.

## Funding

The project was supported by The Czech Science Foundation (GA18-10144S) and Charles University grants program Progress Q36 and Q40 and SVV 260531/SVV/2020 as well as the grant from the Czech Ministry of Health (RVO-VFN64165) and, “Excelles Program/CarDia project as part of the EU Recovery and Resilience Facility funding”.

## Conflict of Interest

The authors declare that the research was conducted in the absence of any commercial or financial relationships that could be construed as a potential conflict of interest.

## Publisher’s Note

All claims expressed in this article are solely those of the authors and do not necessarily represent those of their affiliated organizations, or those of the publisher, the editors and the reviewers. Any product that may be evaluated in this article, or claim that may be made by its manufacturer, is not guaranteed or endorsed by the publisher.
