# Supplementary material for: Hypoxia Induces Saturated Fatty Acids Accumulation and Reduces Unsaturated Fatty Acids Independently of Reverse Tricarboxylic Acid Cycle in L6 Myotubes
Source: Front Endocrinol (Lausanne). 2022 Mar 11;13:663625. doi: 10.3389/fendo.2022.663625 (PMC8963465; doi:10.3389/fendo.2022.663625)

Supplementary material Figure Legends

Figure 1. Lactate Production And Lipid Content After Glutaminase Inhibiton (CB-839 Treatment).
Differentiated L6 myotubes were exposed to hypoxia for 7 days and subsequently treated with 500 nM CB-839 for 24 hours. Lactate **(A)** and total lipid content **(B)** was determined. DMSO was used as a vehicle in control experiments. N=3 in all experiments.

Figure 2. Representative Western Blot Images For Glutaminase, ACLY, SLC38A2 and SLC1A5.
Chemiluminescent signal of glutaminase **(A)**, ATP-dependent citrate lyase **(B)**, SLC38A2 **(C)** and SLC1A5 **(D)** from individual membranes was detected using the ChemiDoc Imaging System (Bio Rad, USA) and subject to densitometric analysis.

Figure 3. Representative Example Of Differentiated And Undifferentiated Cells
Undifferentiated cells aster 2 days in culture **(A)** and differentited L6 cells after 14 days in culture **(B)** were fixed in 10% formaldehyde and stained for 30 min in 1:1000 solution of BODIPY 493/503 (staining lipids) and HOECHST 33258 (staining nuclei). Fluorescence was excited using light of appropriate wave-length and images captured using Leica, SP-5 microscope.

Figure 4. Gene Expression of Glucose Transporters After Diferentiation
Relative gene expression of GLUT-1 and GLUT-4 glucose transporters was determined using qPCR in undifferentiated (2 days in culture) and differentiated cells (14 days in culture) as described in Methods. N=6 for all experiments.

Table 1.

Effect of hypoxia

	PLS-DA	FC log2(FC)			ANOVA				Pattern hunter (Spearman corr. coef r2)		Fold change (power)			Fold change (in %)		
	VIP score	1% vs 12%	4% vs 12%	1% vs 4%	p-value	1% vs 12%	4% vs 12%	1% vs 4%	12->4->1	12/1->4	1% over 12%	4% over 12%	1% over 4%	1% over 12%	4% over 12%	1% over 4%
PG.18.0 18.1..H 8.804 775.54	1,02	1,03	0,31	0,73	2.95E-10	< 0.01	< 0.01	< 0.01			2,042	1,240	1,659	104,20	23,97	65,86
TG.16.0 16.0 16.0..NH4 14.0	1,93	0,80	0,76	0,03	8.88E-08	< 0.01	< 0.01				1,741	1,693	1,021	74,11	69,35	[redacted]
TG.16.0 16.0 18.0..NH4 14.3	2,05	1,14	0,93	0,21	6.07E-08	< 0.01	< 0.01				2,204	1,905	1,157	120,38	90,53	[redacted]
PC.16.0 20.4..H 8.461 782.56	1,39	-1,90	-0,53	-1,38	4.17E-10	< 0.01	< 0.01	< 0.01	-0.95		0,268	0,693	0,384	-73,21	-30,74	-61,58
PC.16.0 22.4..H 9.135 810.59	1,21	-1,64	-0,39	-1,25	6.47E-12	< 0.01	< 0.01	< 0.01	-0.95		0,321	0,763	0,420	-67,91	-23,69	-57,96
PC.16.1 16.1..C2H3O2 8.172	1,01	-0,82	-0,31	-0,51	5.53E-09	< 0.01	< 0.01	< 0.01	-0.95		0,566	0,807	0,702	-43,36	-19,34	-29,78
PE.16.0 20.3..H 9.705 740.52	1,29	-0,75	-0,45	-0,30	4.78E-07	< 0.01	< 0.01	< 0.01	-0.94		0,595	0,732	0,812	-40,54	-26,80	-18,77
LPE.20.3..H 2.649 502.2919	1,14	-0,58	-0,36	-0,22	0.000469	< 0.01	< 0.01		-0.82		0,669	0,779	0,859	-33,10	-22,08	[redacted]
PE.18.0 20.3..H 10.60 768.55	1,08	-0,85	-0,35	-0,50	4.52E-05	< 0.01	< 0.01	< 0.01	-0.87		0,555	0,785	0,707	-44,52	-21,54	-29,29
PE.18.0 20.4..H. 9.736 766.53	1,30	-1,01	-0,51	-0,50	5.37E-07	< 0.01	< 0.01	< 0.01	-0.95		0,497	0,702	0,707	-50,35	-29,78	-29,29
PC.32.2. 8.042 788.5420	1,05	-0,72	-0,32	-0,40	3.18E-07	< 0.01	< 0.01	< 0.01	-0.94		0,607	0,801	0,758	-39,29	-19,89	-24,21
PC.32.2. 8.070 752.5204	1,02	-1,29	-0,24	-1,05	9.79E-09	< 0.01		< 0.01	-0.91		0,409	0,847	0,483	-59,10	[redacted]	-51,70
PC.34.3. 8.151 778.5360	1,04	-1,16	-0,29	-0,87	7.5E-07	< 0.01		< 0.01	-0.91		0,448	0,818	0,547	-55,25	[redacted]	-45,29
PC.34.3. 8.407 778.5363	1,17	-1,51	-0,37	-1,14	6.47E-12	< 0.01	< 0.01	< 0.01	-0.95		0,351	0,774	0,454	-64,89	-22,62	-54,62
PC.38.1. 9.726 838.6293	1,13	-1,25	-0,35	-0,90	1.72E-09	< 0.01	< 0.01	< 0.01	-0.95		0,420	0,785	0,536	-57,96	-21,54	-46,41
PC.38.3. 9.843 834.5978	1,12	-1,15	-0,34	-0,80	1.37E-08	< 0.01	< 0.01	< 0.01	-0.95		0,451	0,790	0,574	-54,94	-21,00	-42,57
PI.18.0 20.3..H 9.191 887.563	1,23	-1,89	-0,36	-1,53	9.89E-13	< 0.01	< 0.01	< 0.01	-0.93		0,270	0,779	0,346	-73,02	-22,08	-65,37
PI.18.1 20.3..H. 8.409 885.54	1,16	-1,15	-0,39	-0,76	1.3E-07	< 0.01	< 0.01	< 0.01	-0.93		0,451	0,763	0,590	-54,94	-23,69	-40,95
Plasmenyl.PE.P.16.0.20.3..H 1	1,31	-0,72	-0,45	-0,27	0.003205	< 0.01	< 0.01				0,607	0,732	0,829	-39,29	-26,80	[redacted]
Plasmenyl.PE.P.16.0.20.4..H.	1,37	-1,21	-0,54	-0,67	0.000274	< 0.01	< 0.01	< 0.01	-0.83		0,432	0,688	0,629	-56,77	-31,22	-37,15
Plasmenyl.PE.P.16.0.22.4..H.	1,12	-0,96	-0,36	-0,60	0.000431	< 0.01		< 0.01	-0.81		0,514	0,779	0,660	-48,59	[redacted]	-34,02
TG.14.0 16.0 18.1..NH4. 13.6	1,49	-0,84	0,55	-1,38	4.32E-09	< 0.01	< 0.01	< 0.01			0,559	1,464	0,384	-44,14	46,41	-61,58
TG.14.0 18.1 18.1..NH4 13.6	1,46	-2,00	0,37	-2,38	8.31E-12	< 0.01	< 0.01	< 0.01	-0.77	0.78	0,250	1,292	0,192	-75,00	29,24	-80,79
TG.16.0 16.0 18.1..NH4. 14.0	1,77	-0,50	0,71	-1,20	8.65E-08	< 0.01	< 0.01	< 0.01		0.87	0,707	1,636	0,435	-29,29	63,58	-56,47
TG.16.0 18.1 18.1..NH4. 14.0	1,54	-1,80	0,51	-2,31	1.59E-10	< 0.01	< 0.01	< 0.01			0,287	1,424	0,202	-71,28	42,41	-79,83
TG.16.0 18.1 18.1..NH4. 14.1	1,45	-1,35	0,48	-1,83	1.7E-09	< 0.01	< 0.01	< 0.01			0,392	1,395	0,281	-60,77	39,47	-71,87
TG.16.0 18.1 20.2..NH4. 13.9	1,49	-2,18	0,36	-2,54	3.29E-11	< 0.01	< 0.01	< 0.01	-0.77		0,221	1,283	0,172	-77,93	28,34	-82,81
TG.16.0 18.1 20.2..NH4. 14.1	1,35	-1,60	0,34	-1,94	1.29E-09	< 0.01	< 0.01	< 0.01			0,330	1,266	0,261	-67,01	26,58	-73,94
TG.18.0 18.1 18.1..NH4. 14.3	1,65	-1,51	0,64	-2,15	1.74E-09	< 0.01	< 0.01	< 0.01			0,351	1,558	0,225	-64,89	55,83	-77,47
PC.15.0 16.0..C2H3O2. 9.208	1,00	-1,21	0,03	-1,24	1.36E-10	< 0.01		< 0.01	-0.84		0,432	1,021	0,423	-56,77	[redacted]	-57,66
PG.18.1 18.1..H 8.794 773.53	1,00	-1,17	0,10	-1,27	1.34E-09	< 0.01		< 0.01	-0.80		0,444	1,072	0,415	-55,56	[redacted]	-58,53
PE.16.0 20.4..H 8.995 738.50	1,07	0,52	-0,27	0,79	0.000857	< 0.01		< 0.01			1,434	0,829	1,729	43,40	[redacted]	72,91
TG.16.1 18.1 18.1..NH4. 13.6	1,44	-2,41	0,20	-2,61	6.47E-12	< 0.01		< 0.01	-0.82		0,188	1,149	0,164	-81,18	[redacted]	-83,62
TG.17.0 17.0 18.1..NH4. 14.3	2,00	-0,25	0,83	-1,08	9.39E-07		< 0.01	< 0.01		0.91	0,841	1,778	0,473	[redacted]	77,77	-52,70
SM.33.1. 8.269 747.5625	1,15	-0,15	0,30	-0,45	0.000264		< 0.01	< 0.01		0.79	0,901	1,231	0,732	[redacted]	23,11	-26,80

Table 2.

	1% vs 1%+SB204990		4% vs 4%+SB204990		12% vs 12%+CS204990		Hypoxia levels	Presence of SB204990	Interaction of both	Fold change (in %)		
	Change	Fold	Change	Fold	Change	Fold	p-value_A	p-value_B	p value_AB	1% O2	4% O2	12% O2
**X1 FA.22.4..H 4.766 331.2651**	DOWNR	1,55	UPREGU	1,11	UPREG	1,12	0,000106987	0,273118494	0,002171433	55	11	12
**X1 LPE.18.0..H 4.098 480.3076**	DOWNR	1,17	DOWNR	1,02	UPREG	1,10	1,58E-12	0,377677865	0,002271444	17	2	10
**X1 PI.16.0 20.4..H 7.714 857.5163**	DOWNR	1,32	DOWNR	1,06	DOWNR	1,09	7,21E-12	5,97E-06	0,002166443	32	6	9
**X1 TG.14.0 16.0 18.1..NH4. 13.69 822.**	UPREG	1,60	UPREGU	1,01	UPREG	1,13	6,12E-13	0,000157311	0,000586797	60	1	13
**X1 TG.14.0 18.1 18.1..NH4 13.69 848.**	UPREG	1,77	UPREGU	1,03	UPREG	1,16	0	7,94E-05	0,000356197	77	3	16
**X1 TG.16.0 16.0 18.1..NH4. 14.02 850.**	UPREG	1,57	UPREGU	1,01	UPREG	1,18	8,75E-11	0,000185786	0,002253208	57	1	18
**X1 TG.16.0 18.1 18.1..NH4. 14.01 876.**	UPREG	1,81	UPREGU	1,02	UPREG	1,16	0	7,56E-05	0,000260935	81	2	16
**X1 TG.16.0 18.1 18.1..NH4. 14.12 876.**	UPREG	1,60	DOWNR	1,01	UPREG	1,10	2,22E-15	0,001996307	0,001816011	60	1	10
**X1 TG.16.0 18.1 20.2..NH4. 13.99 902.**	UPREG	1,74	UPREGU	1,02	UPREG	1,19	0	0,00014327	0,001163189	74	2	19
**X1 TG.16.0 18.1 20.2..NH4. 14.14 902.**	UPREG	1,56	UPREGU	1,02	UPREG	1,11	0	0,001295299	0,004757904	56	2	11
**X1 TG.16.1 18.1 18.1..NH4. 13.68 874.**	UPREG	1,66	UPREGU	1,03	UPREG	1,17	0	0,000347605	0,002634595	66	3	17
**X1 TG.18.0 18.1 18.1..NH4. 14.33 904.**	UPREG	1,80	UPREGU	1,06	UPREG	1,18	1,55E-15	0,000205693	0,002949243	80	6	18
**X3 PC.30.1. 8.094 726.5049**	DOWNR	1,04	DOWNR	1,07	UPREG	1,18	0	0,511485648	0,007358105	4	7	18
**X3 PC.38.4. 9.280 832.5819**	UPREG	1,91	DOWNR	1,11	UPREG	1,11	0,00032634	0,008381067	0,001183333	91	11	11

Figure 1. Lactate Production And Lipid Content After Glutaminase Inhibiton (CB-839 Treatment)

A.

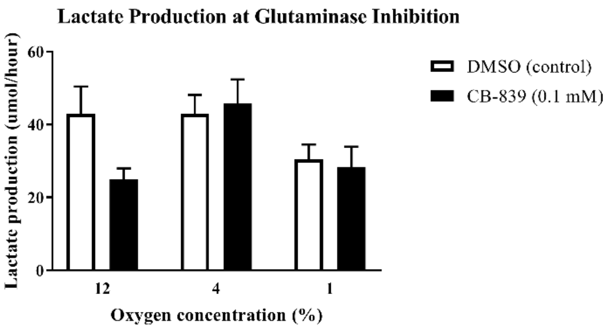

B.

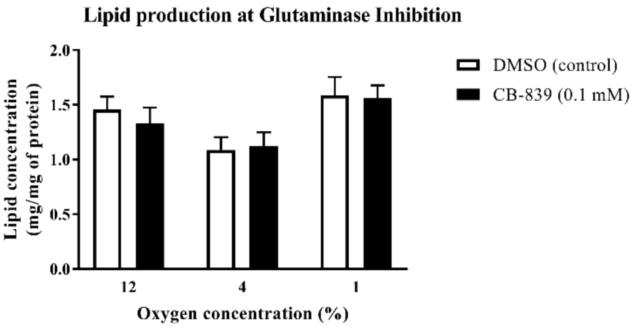

Figure 2. Representative Western Blot Images For Glutaminase, ACLY, SLC38A2 and SLC1A5

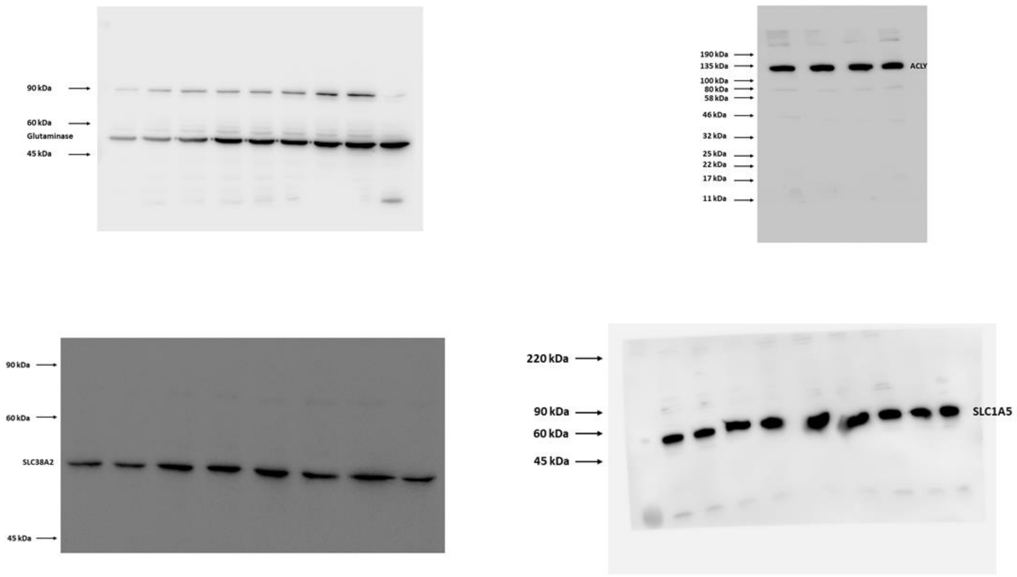

Figure 3. Representative Example Of Differentiated And Undifferentiated Cells

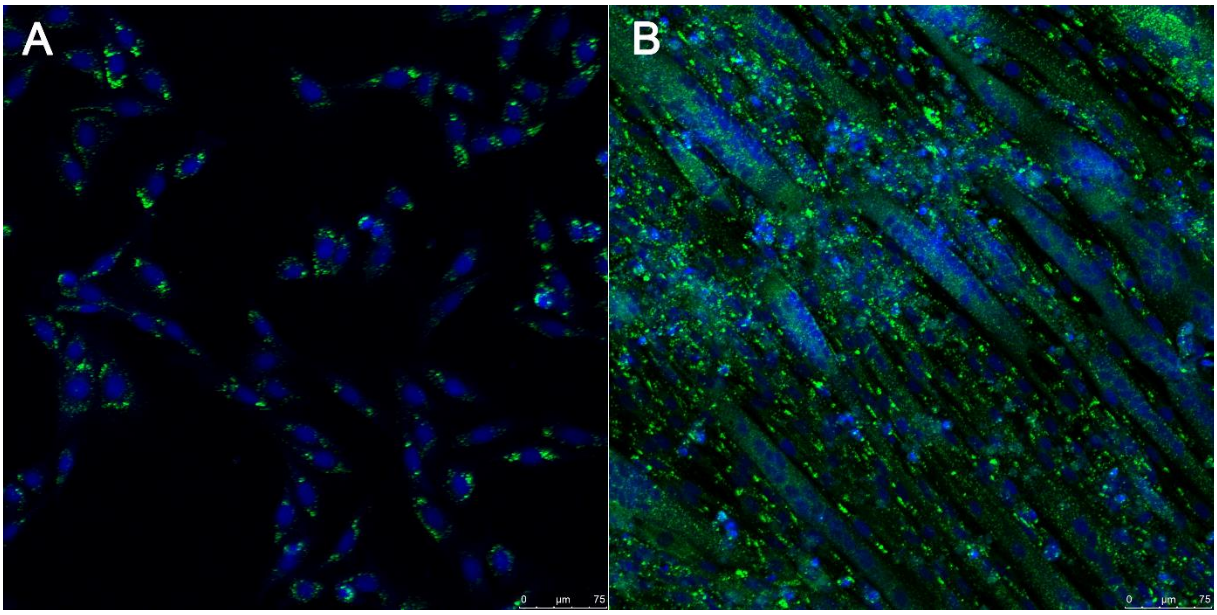

Figure 4. Gene Expression of Glucose Transporters After Diferentiation

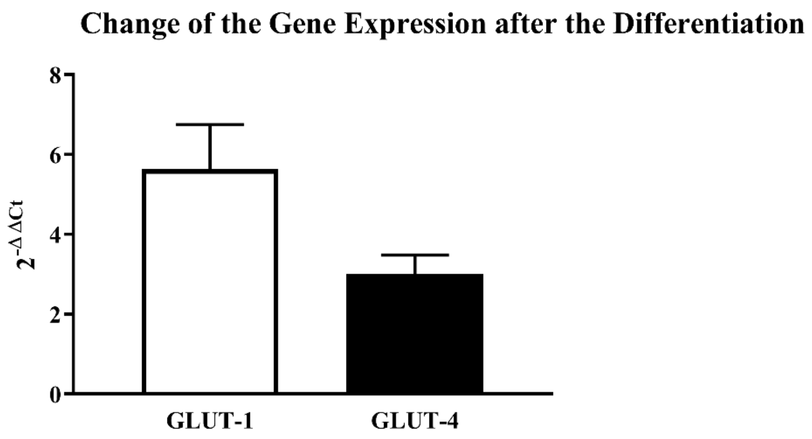

Supplement: Supplementary file 1 [file DataSheet_1.pdf]
